# Supplementary material for: Visual stimulus features that elicit activity in object-vector cells
Source: Commun Biol. 2021 Oct 25;4:1219. doi: 10.1038/s42003-021-02727-5 (PMC8545948; doi:10.1038/s42003-021-02727-5)
Supplement: Supplementary file 2 — Supplementary Information [file 42003_2021_2727_MOESM2_ESM.pdf]

Supplementary Information:

Visual stimulus features that elicit activity in object-vector cells

Sebastian O. Andersson, Edvard I. Moser and May-Britt Moser

Kavli Institute for Systems Neuroscience and Centre for Neural Computation, Norwegian University of Science and Technology (NTNU), Trondheim, Norway

Corresponding authors: Sebastian O. Andersson: sebastian.o.andersson@ntnu.no; May-Britt Moser: may-britt.moser@ntnu.no

Supplementary Figure 1

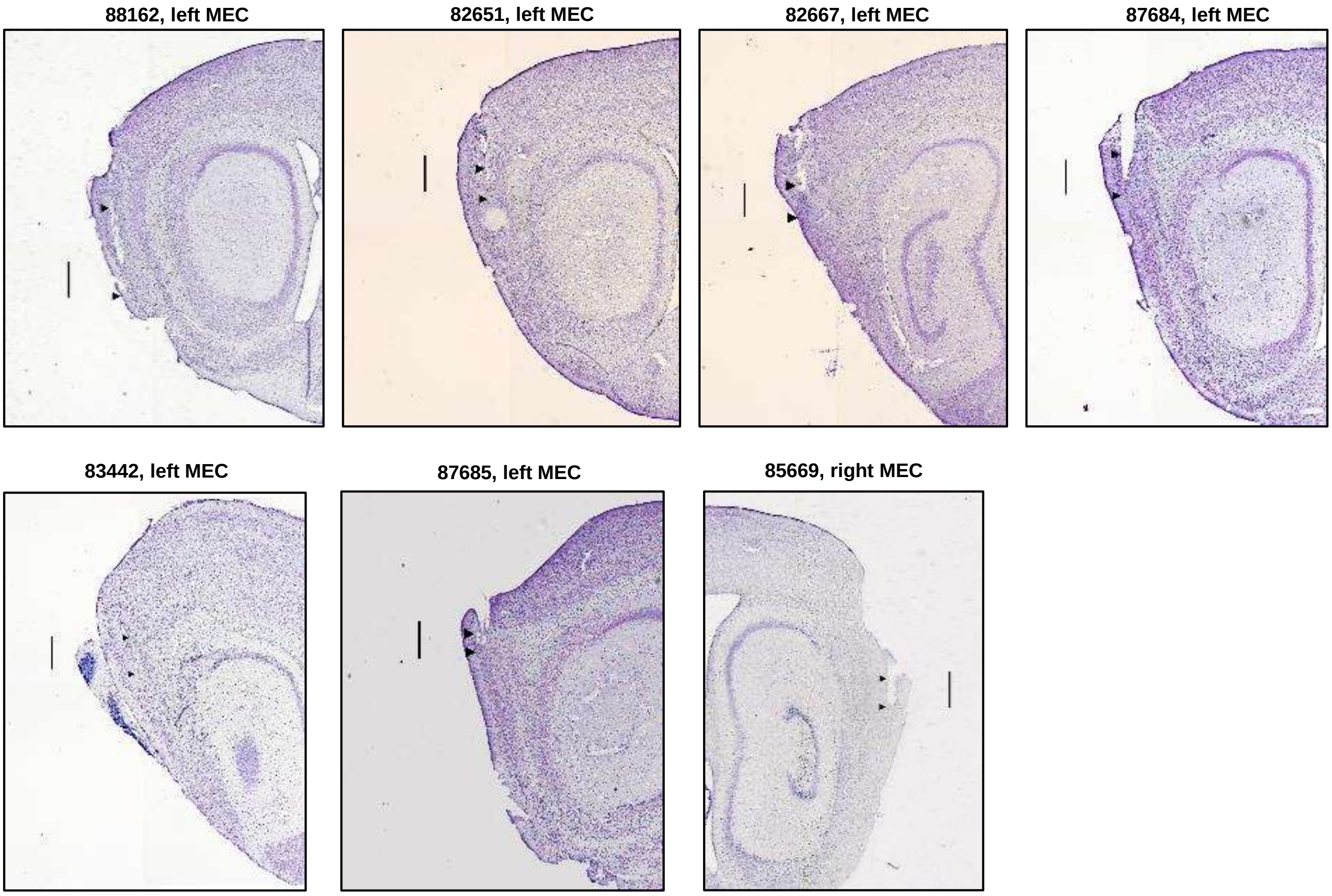

Supplementary Figure 1. Recording locations in the MEC. Nissl-stained sagittal brain sections showing tetrode locations for the 7 mice used in the experiments. Mouse identifier (ID) numbers and brain hemisphere are indicated above each section. Pairs of black arrowheads indicate the dorsoventral range of recording locations in which OV cells were found.

Supplementary Figure 2

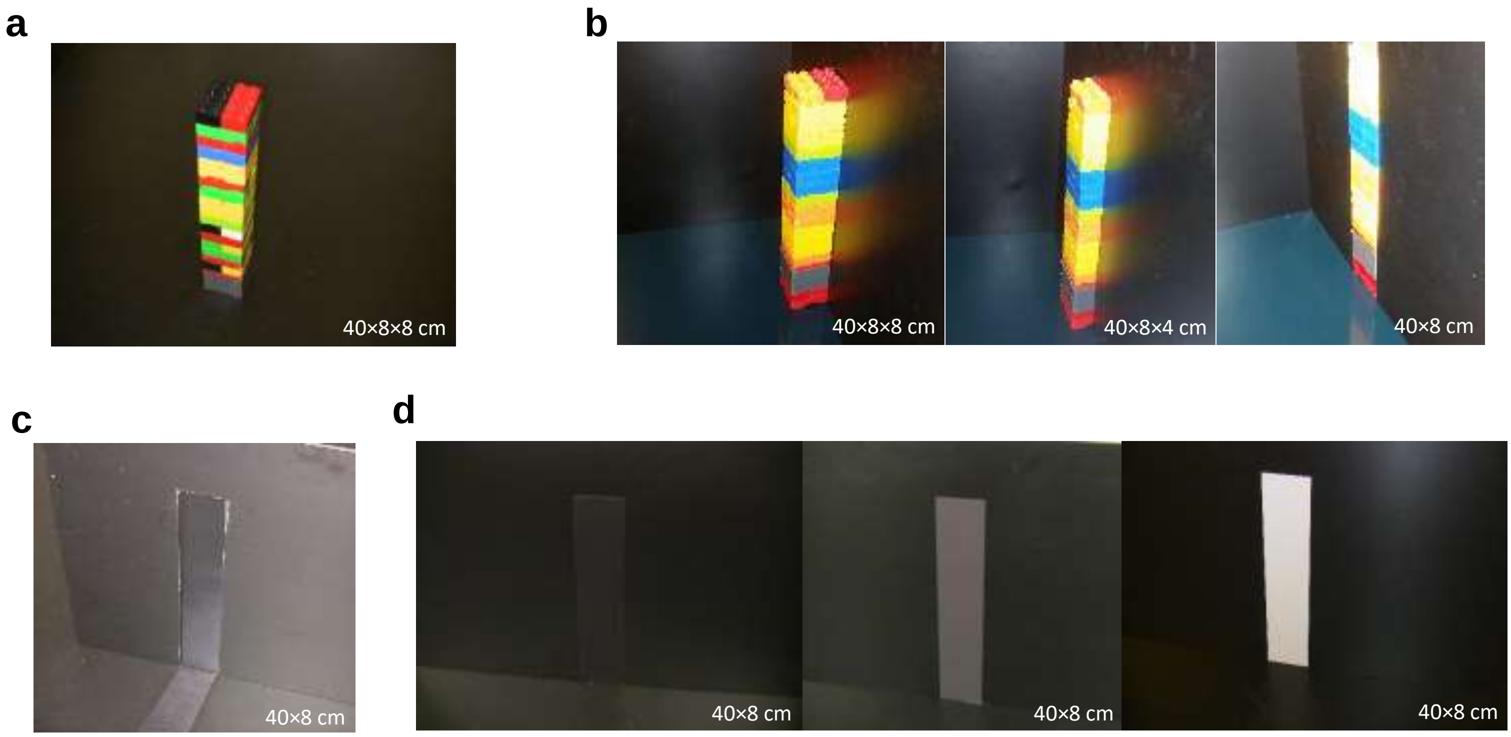

Supplementary Figure 2. Objects used in the experiments. a, Tower-like object made of Duplo used in the ‘Object’ and ‘Moved Object’ trials (Fig. 1a, middle and right) in order to identify OV cells. The object’ size was 40×8×8 cm^3 (height, length, width). **b**, Objects used in the 2D/3D experiment (Fig. 2). Three configurations were tested: in one (left), the full 3D shape of the object was inside the arena, exposing 100% of its volume. In a second configuration (middle), the object was partially embedded into the wall, exposing 50% of its volume. In the third configuration (right), the object was fully embedded into the arena wall, appearing as a visually distinct segment of the otherwise continuous arena wall. **c,** Transparent 2D surface used in the experiment reported in Fig. 3. A transparent film was present on the outside of a 40×8 cm^2 hole in the arena wall. Behind the wall and the opening, the animal could see a uniform surface formed by dark blue curtains surrounding the test arena (located ~1 m away from the arena walls). **d,** Visual contrasts used in the experiment reported in Fig. 4. The visual contrast was a 40×8 cm^2 band of self-adhesive tape printed on the wall, with varying levels of whiteness (10%, 60% and 100%, respectively).

Supplementary Figure 3

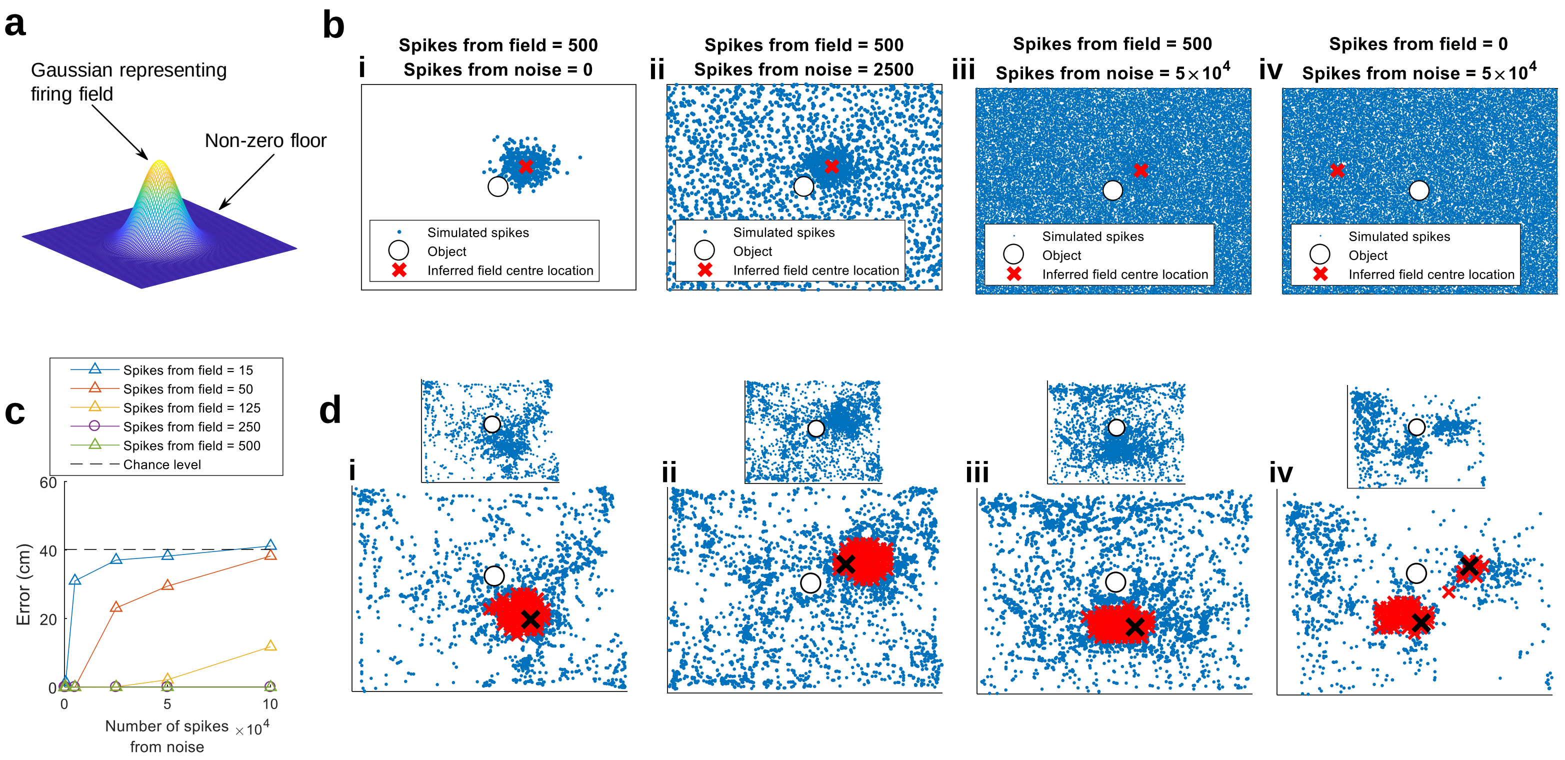

Supplementary Figure 3. Field detection algorithm. a, Firing fields were detected by an algorithm that models the spike data as a mixture of two processes. The first process is a Gaussian representing the cell's firing field. The second process is a uniform floor with non-zero probability (a Gaussian with infinite variance). This represents the fact that the cell might spike anywhere in the environment, regardless of its spatial firing pattern. The second process is the one responsible for clearing up noise. **b**, Representative examples of field detection on simulated data. In the first three examples, the number of spikes drawn from the field is 500, while the number of spikes drawn from noise is varied between 0 (i), 2500 (ii) and $5×10^4$ (iii). The algorithm identifies the correct location of the field in all cases. When only noise is present, as in the last example (0 spikes from the field, $5×10^4$ spikes from noise), the algorithm chooses a random location (iv). **c**, Performance of algorithm with error in Euclidian distance (cm) as a function of the number of spikes drawn from noise (x-axis) and the number of spikes drawn from the field (colored curves). When the number of spikes in the field is 500 or 250, perfect performance is achieved across all noise levels (mean error 0 cm). When the number of spikes in the field is 125, the mean error is less than 2.5 cm for all noise levels except the largest one (mean error 11.8 cm). The error is large only if the field contains 50 spikes or less. Note that 500 spikes from the field is the same as in examples from panel b, i-iii. The data points are means with 50 repetitions for each condition. The dotted line indicates chance level. **d**, Representative examples of field detection on real data. Red crosses show the x, y coordinates with the 50 largest probability values in the algorithm's probability distribution for the fields. Black crosses show the x, y coordinates with the maximum probability for each local cluster. Examples are from OV cells with one field (i-iii) and an OV cell with two fields (iv). The number of fields was determined by the number of local maxima in the probability distribution. Insets show path plots before field detection for clearer visualisation.

Supplementary Figure 4

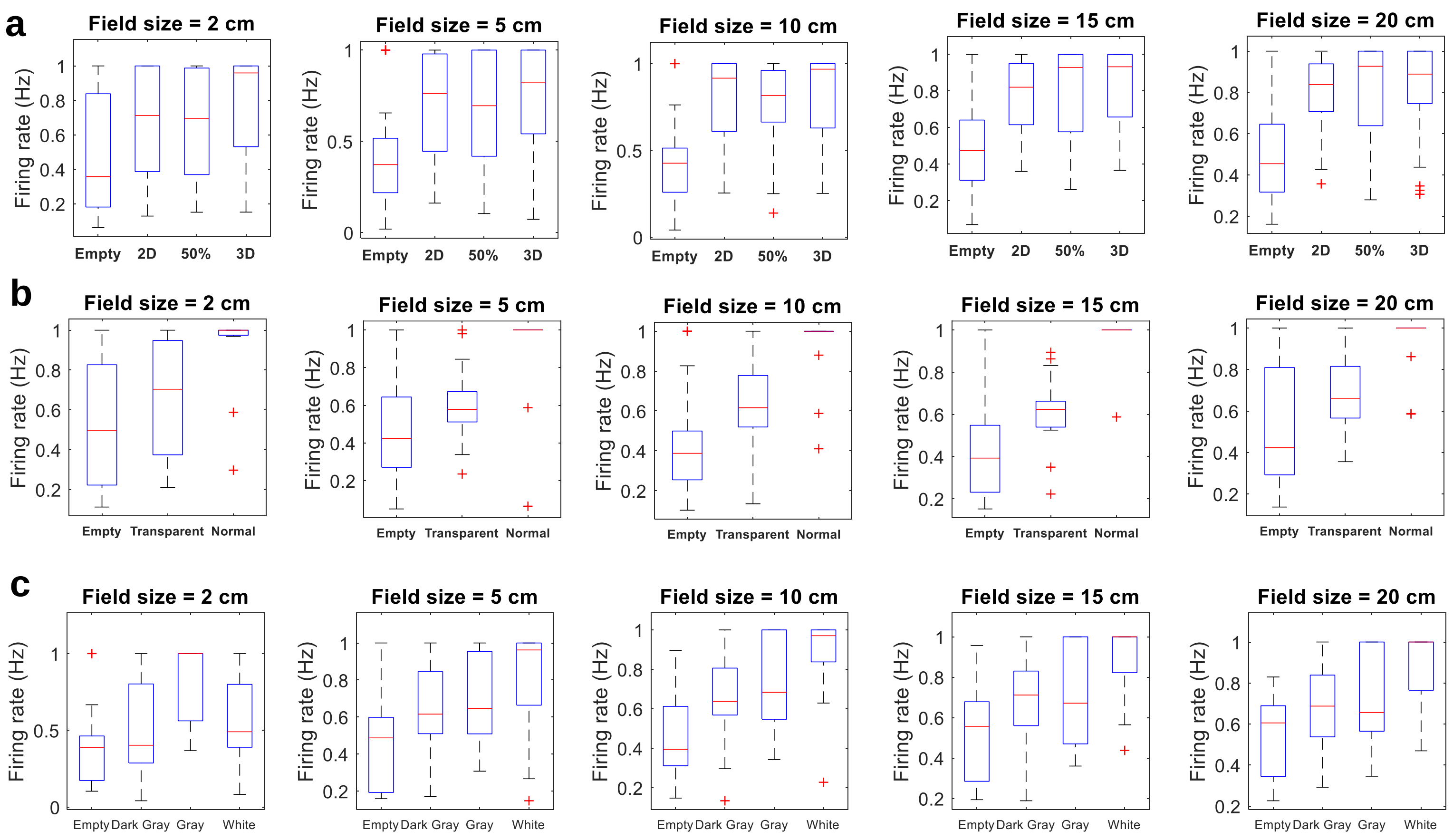

Supplementary Figure 4. Effect of field size parameter on results in Fig. 2, 3 and 4. a, Box-and-whisker plots of normalised firing rate (Hz) of OV cells in the 2D/3D experiment in Fig. 2c, left panel. For each cell, we calculated the firing rate inside an ROI in which we expected the cell to fire based on its vector coordinates. The vector coordinates are found using the Bayesian algorithm described in Supplementary Fig. 3. The size of the ROI (i.e. field size) was a parameter set by the experimenter. Each panel shows a different field size (2, 5, 10, 15 and 20 cm). Note that the pattern of results is the same regardless of the value of the field size parameter. **b**, Same as in the previous panel but box-and-whisker plots from the transparent-object experiment corresponding to Fig. 3c, left panel. Note that the pattern of results is the same regardless of field size. The data in the 'Normal' condition have little or no variation because nearly all cells have their maximum response to this object, which after normalisation yields a value of 1 for every cell. **c,** Same as in the previous panels but box-and-whisker plots from the contrast experiment corresponding to Fig. 4c, left panel. Again, the pattern of results is the same regardless of the field size, with the possible exception of the smallest field size (2 cm) in which the gray contrast rather than the white contrast has the strongest increase in firing rate.

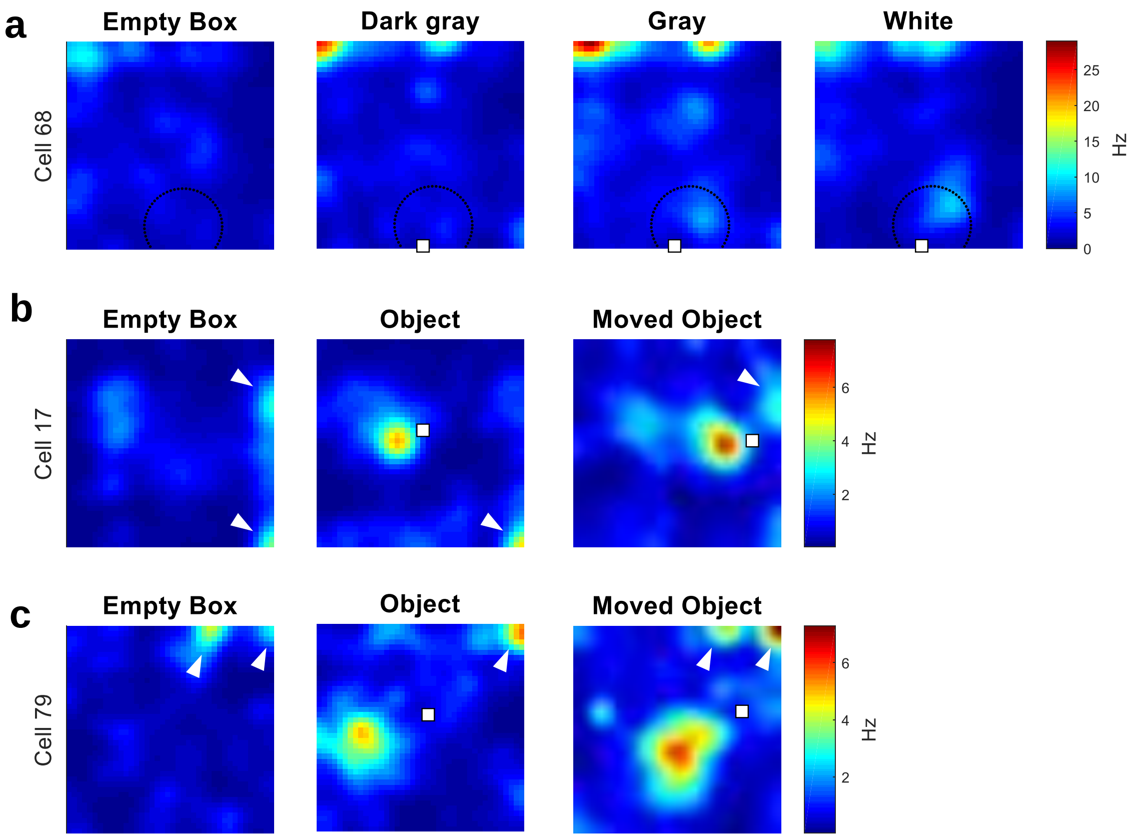

Supplementary Figure 5. Examples of OV cells with weak firing fields, only some of which are induced by the experimental object. a, Example of an OV cell in the contrast experiment (Fig. 4) where the change in contrast of the experimental object elicits a weak increase in firing rate that is difficult to detect by eye. Rate maps show colour-coded firing rate in Hz as a function of the animal's position. The white square marks the object location. The dotted circle marks the ROI in which we expected the cell to fire based on its vector coordinates. The vector coordinates of the cell were found by applying the algorithm described in Supplementary Fig. 3 to the 'Object' trial in which we originally identified the cell as an OV cell (Fig. 1a, middle). **b**, Example of an OV cell with weak firing fields that are not induced by the presence of the object. White arrows indicate the weak firing fields. Given the results from Fig. 4, such weak firing fields might reflect responses to edges, corners, shadows or other types of contrast (see Discussion). Rate map conventions are as in panel a. **c**, Further example of an OV cell with weak firing fields that are not induced by the presence of the object. White arrows indicate the weak firing fields. Rate map conventions are as in panel a. It is notable that in both panels b and c several of the weak firing fields line up with the corners of the recording box. Also note that in both panels, one weak firing field disappears when introducing the object (compare 'Empty Box' and 'Object'), raising the possibility that weak firing fields become inhibited by more salient objects when these are present in the environment.

Supplementary Figure 6

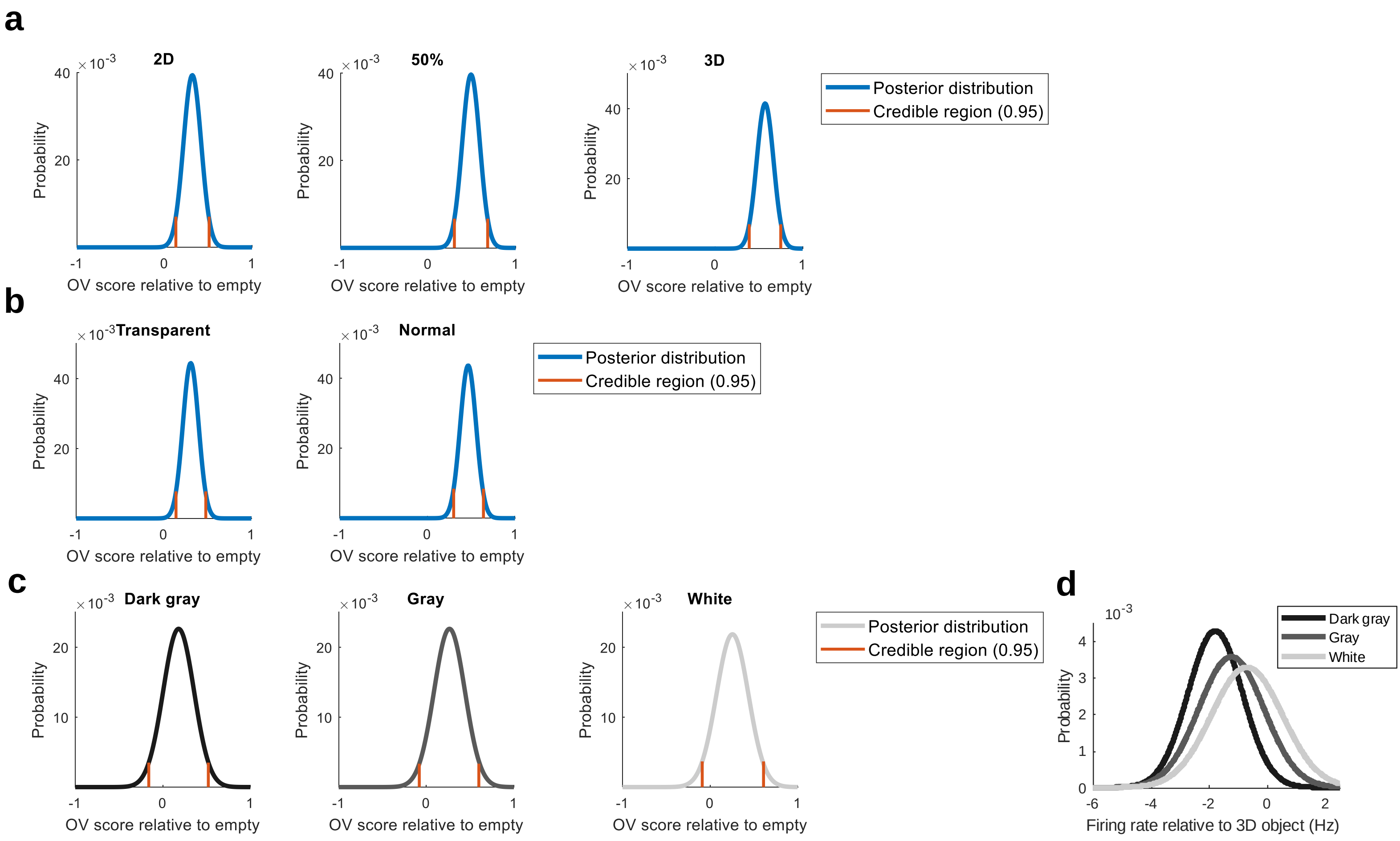

Supplementary Figure 6. Posterior distributions of the increase in OV score to each object or feature. Panels are similar to Fig. 5 but use the OV score rather than the firing rate as input to the Bayesian inference. The OV score in the 'Empty Box' trial has been subtracted so that probability on the right (left) of 0 should be interpreted as a positive (negative) response to the object or feature. Orange bounds represent the credible region, which is the smallest possible region containing 95% of the probability mass. The credible region can be interpreted *as "there is a 95% probability that the increase in the OV score lies between these bounds"*. **a,** Posterior distributions show the probability of the amount of increase in the OV score. The plots correspond to Fig. 2d and show the results for the 2D surface (left), the partially embedded object (middle) and the 3D object (right). **b**, Same as in panel a but posterior distributions from the transparent-object experiment in Fig. 3d. The results are shown for the transparent object in the wall (left) and the freestanding 3D object (right). **c,** Same as in panel a but posterior distributions from the contrast experiment in Fig. 4d. The results are shown for the dark gray contrast (left), the gray contrast (middle) and the white contrast (right). **d,** Probability distributions from the contrast experiment overlaid, plotting the probability of different firing rate changes (Hz) relative to the 3D object. The panel is analogous to Fig. 5e but uses the 3D object rather than the 'Empty Box' trial as reference. Because all contrasts produce weaker activity in OV cells than the 3D object, all probability distributions lie to the left of the origin. The overall trend (increasing probability towards more positive firing rates as a function of contrast) is the same. The trial with the 3D object was the 'Moved Object' trial.

Supplementary Figure 7

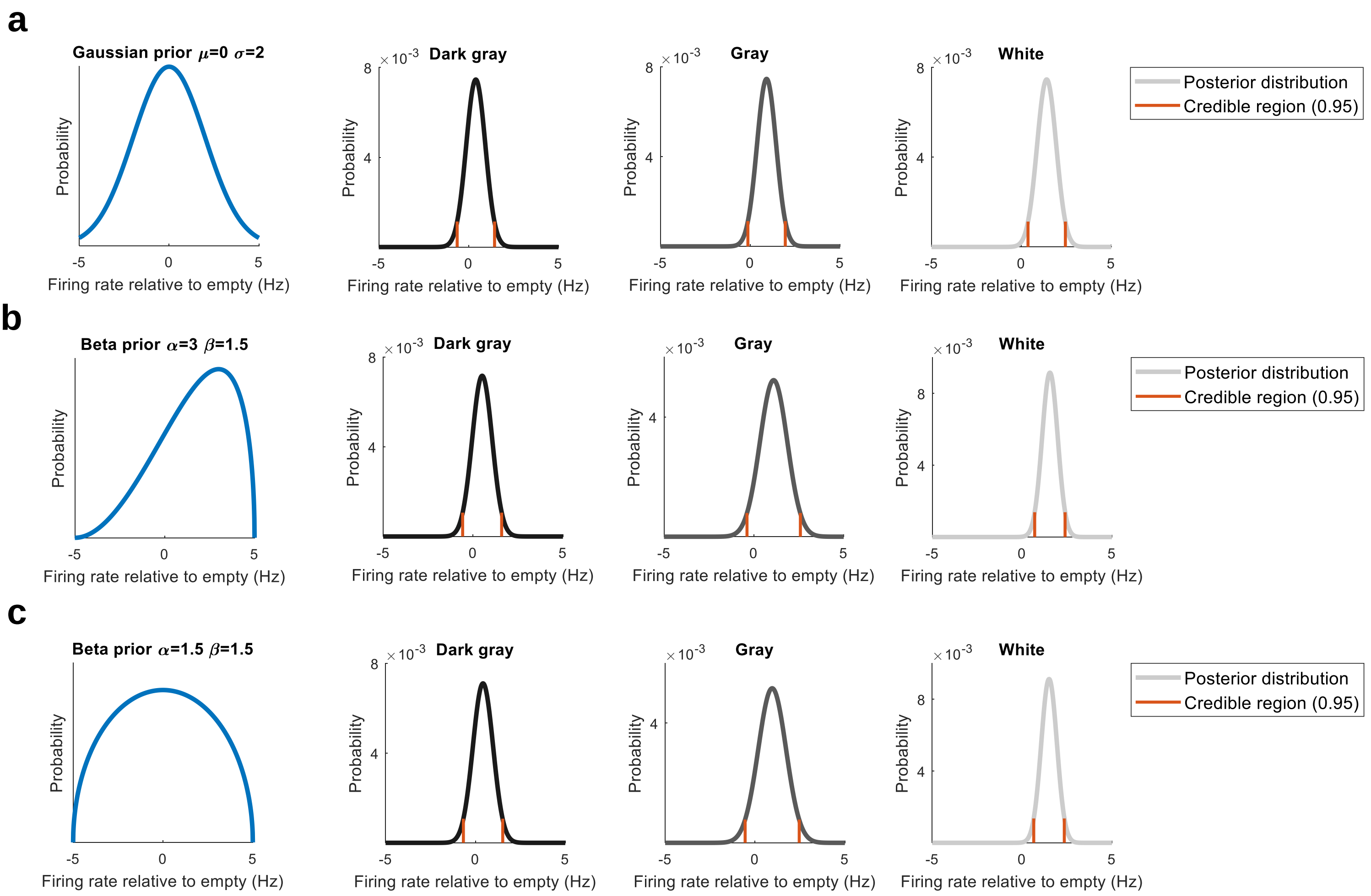

Supplementary Figure 7. Sensitivity of results to different priors. a, Posterior distributions, as in Figure 5d, showing the probability that OV cells respond with any average firing rate to each visual contrast. Instead of a uniform prior, we use a Gaussian prior peaked at 0 with a standard deviation of 2. The results from choosing this prior are effectively identical to the results in Fig. 5d. **b,** Same as in panel a but using a beta prior with parameters alpha=3, beta=1.5 in order to bias firing rates towards positive values. This intuitively corresponds to prior information that OV cells are more likely to respond with a higher firing rate when an object is present in the environment, pointing to this prior as a possible alternative to the more conservative uniform prior chosen in this study. The three panels to the right show that the results from choosing the prior in b are effectively identical to the results in Fig. 5d. **c**, Same as in panel a but using a beta prior with parameters alpha=1.5, beta=1.5 to admit a wide range of possible values but discount strong changes in FR. The results from choosing this prior are effectively identical to the results in Figure 5d. Overall, the results from panels a, b and c confirm that the amount of data we have obtained is sufficient because any reasonable prior gives identical results.

Supplementary Figure 8

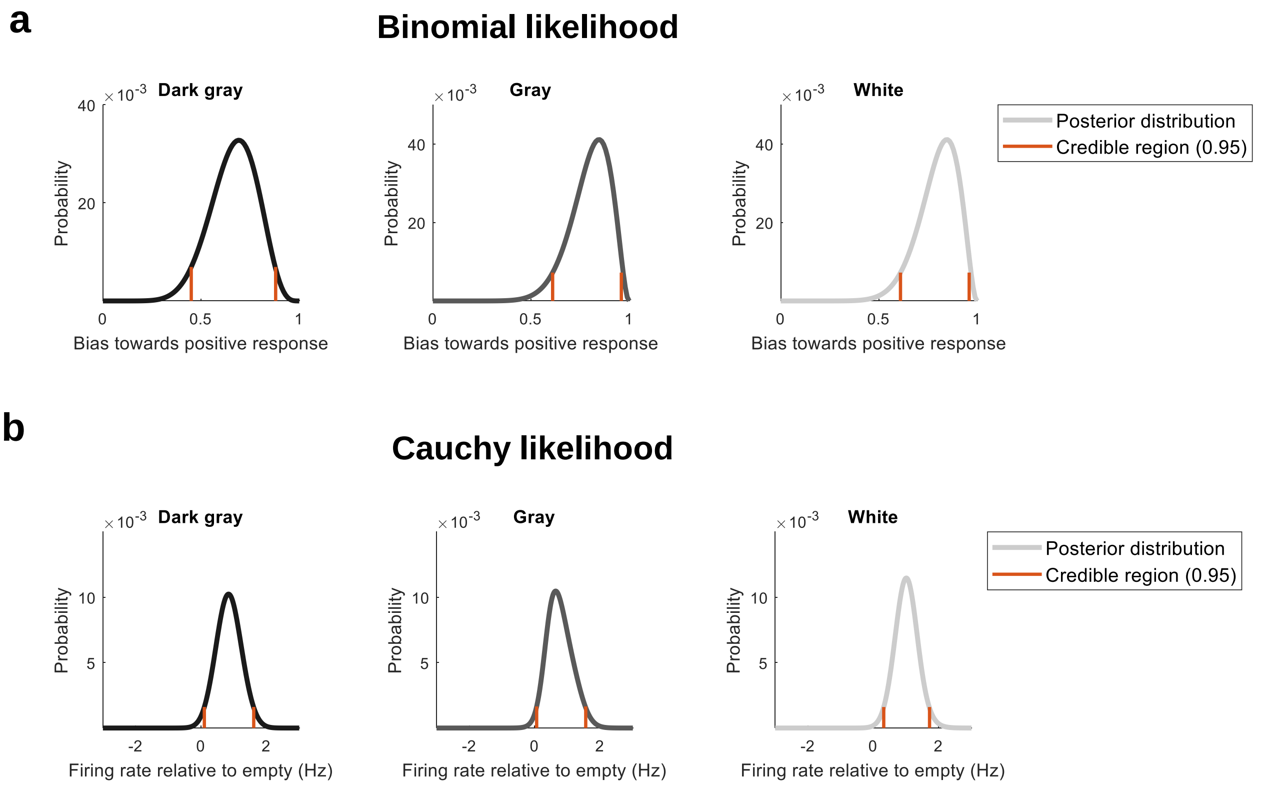

Supplementary Figure 8. Sensitivity of results to different likelihoods. a, Bayesian inference using a binomial likelihood instead of a Gaussian likelihood. Here, we have binarized the data into either response (x = 1) or no response (x = -1) by applying the sign function to every data point (which gives x=1 if FR is > 0 Hz and x=-1 if FR is < 0 Hz). This divides the data into r successes and (n - r) failures. n is the total number of OV cells we have data from, r is the number of OV cells that respond with a firing rate change > 0 Hz and n - r is the number of OV cells that respond with a firing rate change < 0 Hz. We then infer the 'bias' of OV cells to respond to the object or feature. Note that this is equivalent to inferring the bias of a coin after tossing the coin n times, observing r heads and n - r tails. The posterior distributions of the bias of OV cells are shown for the dark gray contrast (left), the gray contrast (middle) and the white contrast (right). Note that as the visual contrast increases, the bias towards a positive response increases, consistent with the results presented in Fig. 5d and e. **b,** Bayesian inference using a Cauchy likelihood instead of a Gaussian likelihood. The Cauchy distribution is a unimodal distribution with fatter tails than a Gaussian. Posterior distributions show the probability that OV cells respond with any average firing rate to each feature. The posterior distributions are shown for the dark gray contrast (left), the gray contrast (middle) and the white contrast (right). The results are similar to the results in Fig. 5d and e. The main difference is that here the responses to the dark gray and gray contrasts also have a high probability of being above 0 Hz. In contrast, with the Gaussian likelihood, there was a non-negligible amount of probability mass below 0 Hz for both the dark gray and gray contrasts (Fig. 5d and e). Thus, the overall pattern of results is the same (or stronger) when using a Cauchy distribution and confirms that our conclusions are robust to using likelihood functions with widely different tails (fat tails in the case of the Cauchy distribution; light tails in the case of the Gaussian distribution).

Supplementary Figure 9

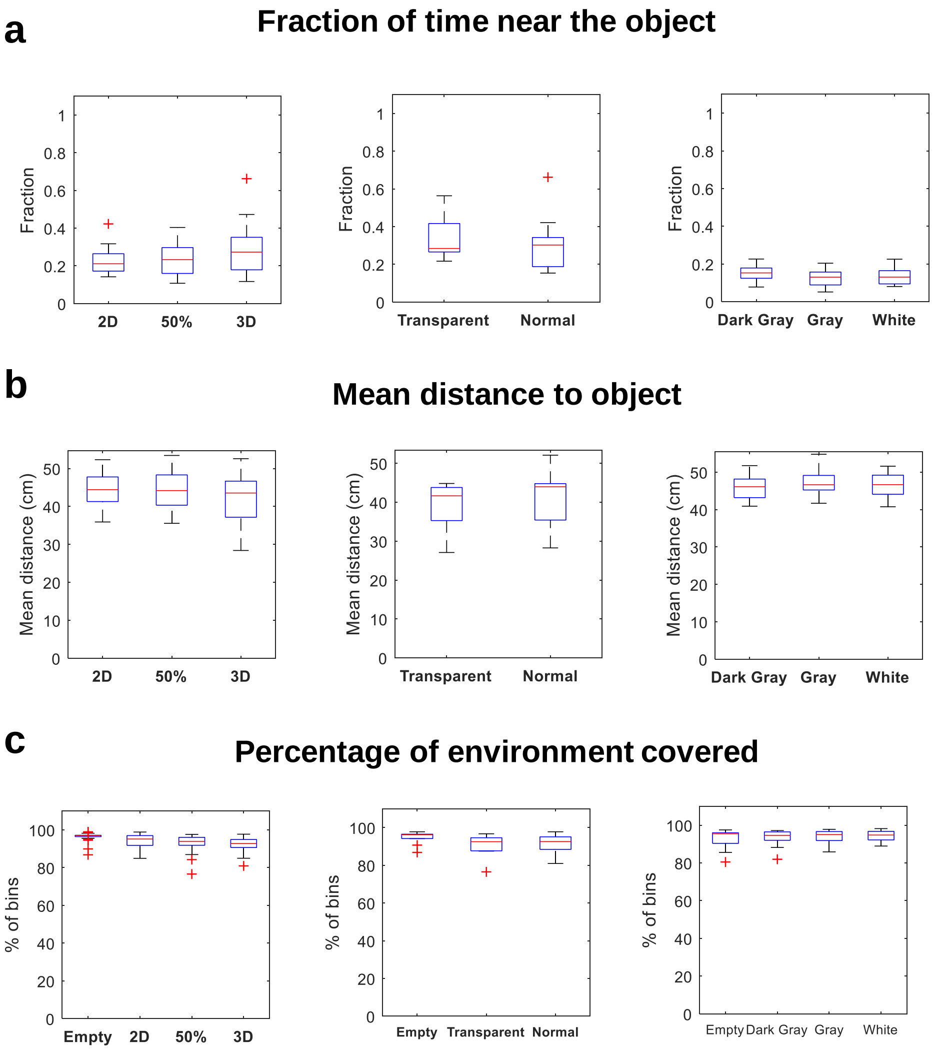

Supplementary Figure 9. Behaviour of mice for different object types. a, Box-and-whisker plots of the fraction of time that the animal spent near the object. We defined 'near' as position samples in which the animal was less than 25 cm away from the object. Each data point is the behaviour of a mouse in a single trial (n = 26 trials for the 2D/3D experiment; n = 11 trials for the transparent-object experiment; n = 16 trials for the contrast experiment). Within each experiment, the animals spend a roughly equal amount of time near the object. The possible exception is that the distribution for the transparent object is elevated compared to the one for the normal object (middle). Note that this does not correlate with the firing properties of OV cells since they respond stronger to the normal object than the transparent object (Fig. 5c). Across experiments, animals spend somewhat less time near the visual contrasts than the other objects. **b,** Box-and-whisker plots of the average distance of the animal to the object. Each data point is the mean distance of a mouse in a single trial. As in **a**, the average distance is similar for different objects. **c**, Box-and-whisker plots of the percentage of the environment that the animal explored. Each data point is the percentage of spatial bins (2×2 cm) covered by the mouse in a single trial. Note that the percentage of the environment that the mouse explored is high regardless of the object type (median always > 92%).

Supplementary Figure 10

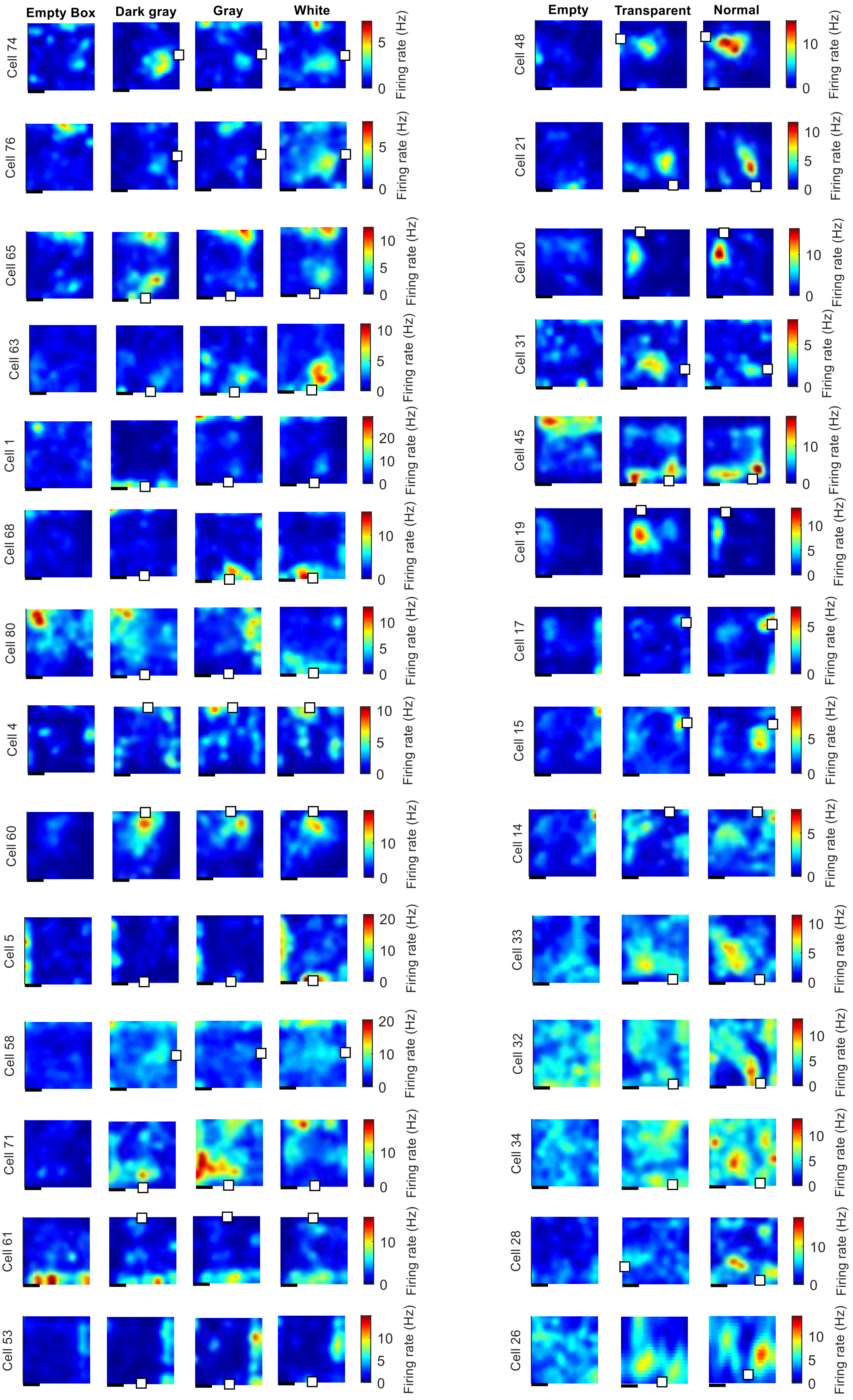

Supplementary Figure 10. Rate maps from OV cells recorded in the contrast experiment and transparent-object experiment. a, Colour-coded rate maps from the contrast experiment. The experiment consisted of four trials: ‘Empty Box’, ‘Dark gray’, ‘Gray’ and ‘White’. Rate maps show colour-coded firing rate in Hz as a function of the animal’s position. The white square marks the object location. Each row represents one cell. For some cells, e.g. cell 74, 76, 1, 63 and 60, the response to visual contrast was clear. For other cells, e.g. cell 58 and 68, the response to visual contrast was only very faint. **b,** Same as in the previous panel but rate maps from the transparent-object experiment. The experimented consisted of three trials: ‘Empty Box’, ‘Transparent’ and ‘Normal’. For the majority of cells, the responses to both the transparent object and normal object are clear. Scale bar, 40 cm.

Supplementary Figure 11

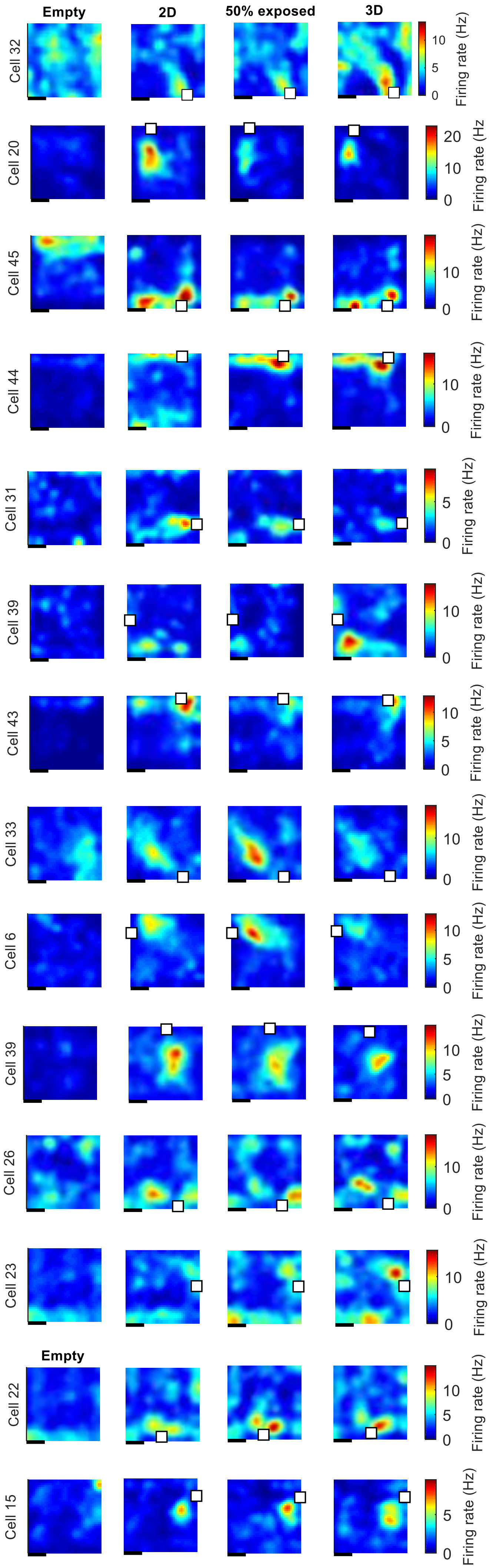

Supplementary Figure 11. Rate maps from OV cells recorded in the 2D/3D experiment. Colour-coded rate maps from the 2D/3D experiment. The experiment consisted of four trials: ‘Empty Box’, ‘2D’, ‘50% exposed’ and ‘3D’. Rate maps show colour-coded firing rate in Hz as a function of the animal’s position. The white square marks the object location. Each row represents one cell. 14 out of 30 recorded cells from the experiment are shown. Scale bar, 40 cm.

Supplementary Figure 12

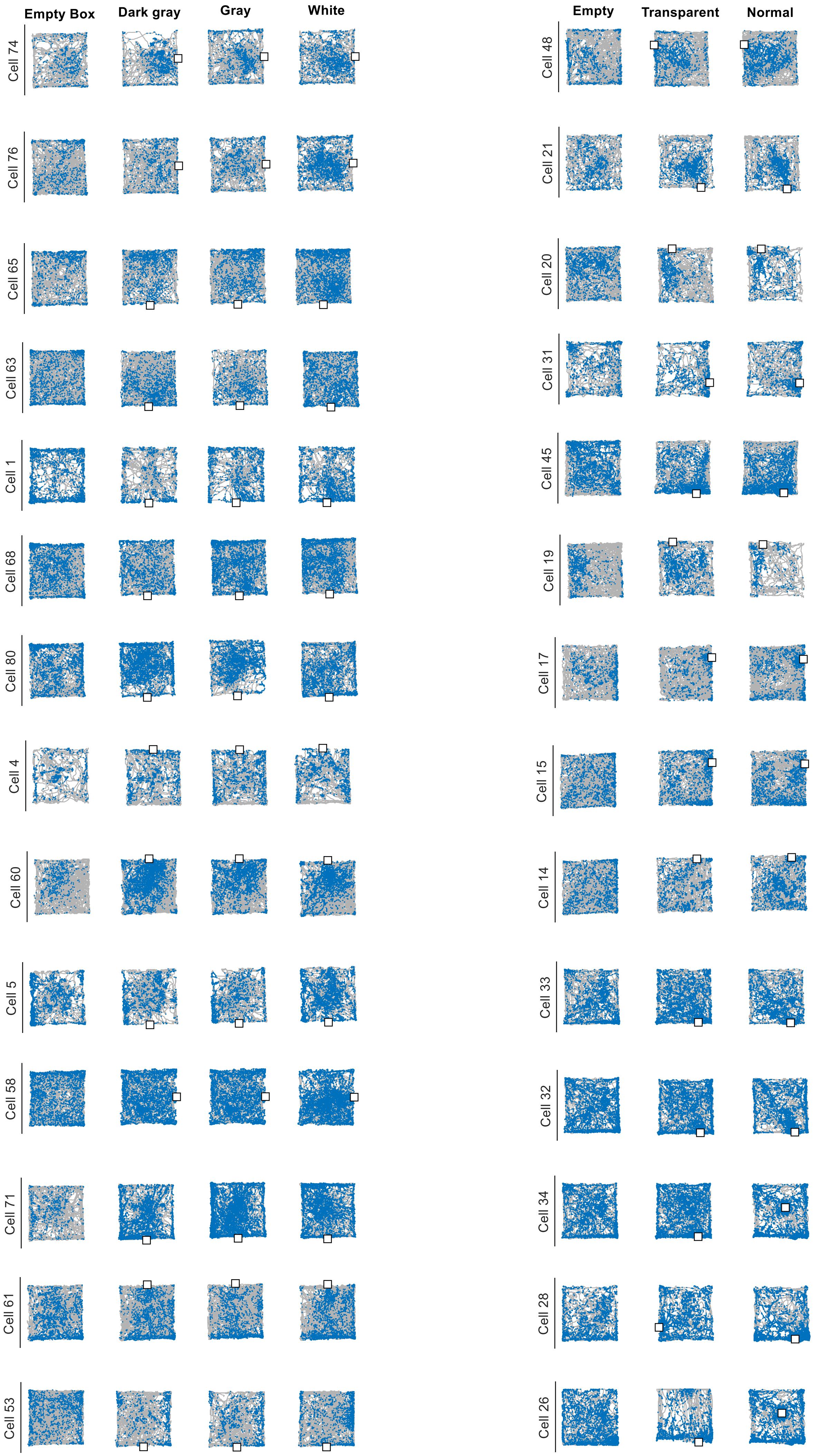

Supplementary Figure 12. Spike plots from OV cells recorded in the contrast experiment and transparent-object experiment. a, Spike plots from the contrast experiment. Blue dots represent spikes, shown overlaid on the animal's trajectory (gray line). The white square marks the object location. **b**, Same as in the previous panel but spike plots from the transparent-object experiment.

Supplementary Figure 13

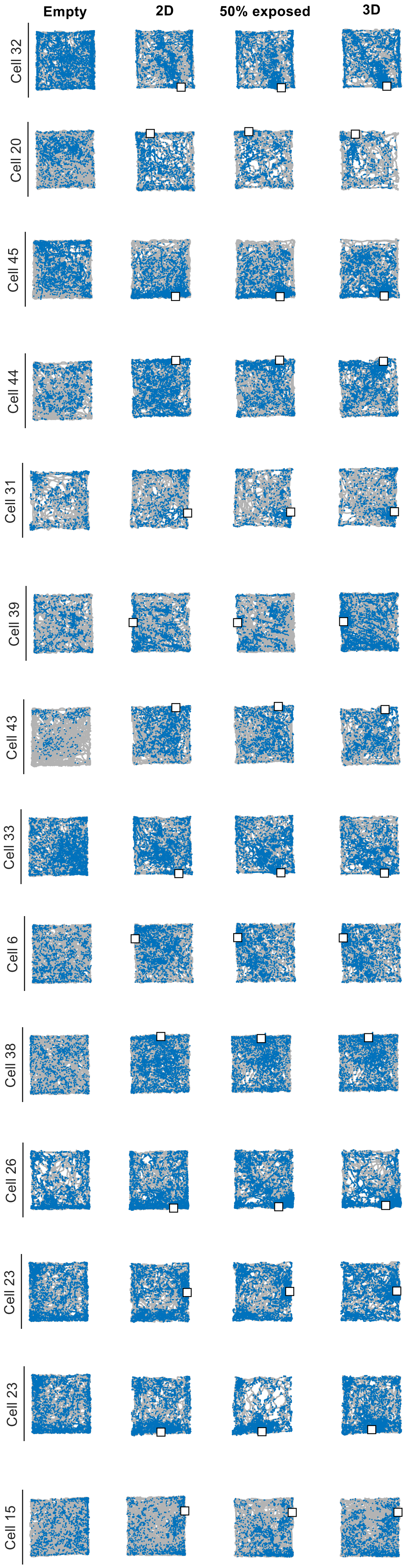

Supplementary Figure 13. Spike plots from OV cells recorded in the 2D/3D experiment. Blue dots represent spikes, shown overlaid on the animal's trajectory (gray line). The white square marks the object location. 14 out of 30 recorded cells from the experiment are shown.

Animal name	Total cell count	OV cell count	OV cell percentage	OV cells in 2D/3D experiment	OV cells in transparent experiment	OV cells in contrast experiment
82651	78	13	16.7%	9	2	
82667	82	13	15.9%	11	6	3
83442	85	12	14.1%	9	6	
87685	35	2	5.7%	1		
88162	106	14	13.2%			7
87684	73	8	11.0%			3
85669	33	5	15.2%			1
Sum	492	67	13.6%	30	14	14

Supplementary Table 1. Cell counts for the different animals. Shown are the total number of cells (2nd column), total number of OV cells and percentages (3rd and 4th column) and number of OV cells in each experiment (5th, 6th and 7th column). Note that since (1) an OV cell can participate in multiple experiments and (2) some OV cells did not participate in any experiments, summing over columns 5, 6 and 7 will not give the counts in column 3.
