# Supplementary material for: Visual stimulus features that elicit activity in object-vector cells
Source: Commun Biol. 2021 Oct 25;4:1219. doi: 10.1038/s42003-021-02727-5 (PMC8545948; doi:10.1038/s42003-021-02727-5)
Supplement: Supplementary file 3 — Description of Additional Supplementary Files [file 42003_2021_2727_MOESM3_ESM.pdf]

Description of Additional Supplementary Files

File name: Supplementary Data 1

Description: Source data for reproducing all graphs and charts in the main figures.
